# Supplementary material for: geodl: An R package for geospatial deep learning semantic segmentation using torch and terra
Source: PLoS One. 2024 Dec 5;19(12):e0315127. doi: 10.1371/journal.pone.0315127 (PMC11620637; doi:10.1371/journal.pone.0315127)
Supplement: Attachment — Submitted filename: RESPONSE TO REVIEWERS.docx [file pone.0315127.s001.docx]

**RESPONSE TO REVIEWERS**

**OVERVIEW**

Thanks for reviewing our manuscript. Note that the editor’s decision was rejection; however, it was suggested that we resubmit following the package’s acceptance to the Comprehensive R Archive Network (CRAN). Thus, this manuscript is being treated as a new submission as opposed to a revised submission. However, we have been asked to respond to the reviewers’ comments and provide a tracked changes document highlighting our edits.

**SUMMARY OF REVISIONS**

The package has now been accepted to CRAN: <https://cran.r-project.org/web/packages/geodl/index.html>.

We have also built a website for the package with associated articles/vignettes and references: <https://wvview.org/geodl/index.html>.

The package website and CRAN page provide ten vignettes/articles that demonstrate the use of the package.

In the revised manuscript we have tried to clearly articulate the purpose of the paper:

*This article has two primary purposes. First, it provides an introduction to the geospatial semantic segmentation workflow implemented by geodl and introduces reference materials and documentation to help researchers and analysts implement the package for their own needs. Second, it provides a detailed documentation of the package’s functions and design philosophy, including the configuration of the provided UNet architectures and their associated modules and the implementation and parameterization of the provided loss and assessment metrics. For specific code-based examples, please see the package webpage and associated vignettes.*

We have also introduced the associated webpages and documentation:

*The geodl package is available on CRAN (*[*https://cran.r-project.org/web/packages/geodl/index.html*](https://cran.r-project.org/web/packages/geodl/index.html)*) and can be installed in R using install.packages(). The source code is available on GitHub (*[*https://github.com/maxwell-geospatial/geodl*](https://github.com/maxwell-geospatial/geodl)*), and the package can alternatively be installed from GitHub using the remotes package [46]. Please consult the following page for details on how to install torch and its dependencies:* [*https://torch.mlverse.org/docs/articles/installation*](https://torch.mlverse.org/docs/articles/installation)*. This page also links to documentation for configuring the environment for GPU-based computation, which is necessary to train CNN-based semantic segmentation models. The package’s official website is available at* [*https://wvview.org/geodl/index.html*](https://wvview.org/geodl/index.html)*. The package webpage complements the material presented here by providing code and detailed vignettes demonstrating the package’s workflow and associated functions. The data required to implement the examples have been provided via FigShare [47]:* [*https://figshare.com/articles/dataset/geodl_example_data/23835165*](https://figshare.com/articles/dataset/geodl_example_data/23835165)*. A separate and smaller version of the data without the image chips used in the training workflow examples is also available on FigShare [48]:* [*https://figshare.com/articles/dataset/geodl_example_data_no_chips_/26824909*](https://figshare.com/articles/dataset/geodl_example_data_no_chips_/26824909)*. More general torch examples are provided on the torch website:* [*https://torch.mlverse.org/*](https://torch.mlverse.org/)*.*

Below, the reviewers’ comments are provided in **BOLD** **BLACK** text. Our comments are provided in red text. Sections extracted from the paper are *red and* *italicized*.

We have tried our best to address the reviewers’ comments. We are willing to make additional edits if we are offered the chance for revision.

**REVIEWERS COMMENTS**

**Reviewer's Responses to Questions**

**Comments to the Author

1. Is the manuscript technically sound, and do the data support the conclusions?

The manuscript must describe a technically sound piece of scientific research with data that supports the conclusions. Experiments must have been conducted rigorously, with appropriate controls, replication, and sample sizes. The conclusions must be drawn appropriately based on the data presented.**

**Reviewer #1: No**

**Reviewer #2: Yes**

**Reviewer #3: Yes**

**Reviewer #4: Yes**

**Reviewer #5: Partly**

**2. Has the statistical analysis been performed appropriately and rigorously?**

**Reviewer #1: N/A**

**Reviewer #2: N/A**

**Reviewer #3: Yes**

**Reviewer #4: Yes**

**Reviewer #5: Yes**

**3. Have the authors made all data underlying the findings in their manuscript fully available?

The** [**PLOS Data policy**](http://www.plosone.org/static/policies.action#sharing) **requires authors to make all data underlying the findings described in their manuscript fully available without restriction, with rare exception (please refer to the Data Availability Statement in the manuscript PDF file). The data should be provided as part of the manuscript or its supporting information, or deposited to a public repository. For example, in addition to summary statistics, the data points behind means, medians and variance measures should be available. If there are restrictions on publicly sharing data—e.g. participant privacy or use of data from a third party—those must be specified.**

**Reviewer #1: No**

**Reviewer #2: Yes**

**Reviewer #3: Yes**

**Reviewer #4: Yes**

**Reviewer #5: Yes**

**4. Is the manuscript presented in an intelligible fashion and written in standard English?

PLOS ONE does not copyedit accepted manuscripts, so the language in submitted articles must be clear, correct, and unambiguous. Any typographical or grammatical errors should be corrected at revision, so please note any specific errors here.**

**Reviewer #1: Yes**

**Reviewer #2: Yes**

**Reviewer #3: Yes**

**Reviewer #4: Yes**

**Reviewer #5: Yes**

**5. Review Comments to the Author

Please use the space provided to explain your answers to the questions above. You may also include additional comments for the author, including concerns about dual publication, research ethics, or publication ethics. (Please upload your review as an attachment if it exceeds 20,000 characters)**

**REVIEWER 1**

**Review of "geodl: an R package for geospatial..." by A. E. Maxwell

The manuscript was, as far as I could tell, a coherent introduction to
a new R package that fills a genuine need. It was was generally well-
structured and written. I am broadly supportive of work such as this
that presents software that accomplishes a difficult task in an
open-source format.**

Thanks for reviewing our manuscript.  **However, the package was far from clean under R CMD check, and for me
this is a show-stopper. There were various errors and warnings that
effectively bar the package from upload to CRAN, and many
organizations simply would not allow anyone to use software that fails
QC in this way.**

Thanks. The software has now been published to CRAN: <https://cran.r-project.org/web/packages/geodl/index.html>

We have also built a website for the package with associated vignettes and references: <https://wvview.org/geodl/index.html> **I was unable to run many of the examples in the Rd files. File
README.Rmd was unfinished and included boilerplate rstudio text;
anyway it did not knit correctly. R CMD Rd2pdf gives a poorly typeset
document.**

These issues were corrected during our CRAN upload/review process. Thanks.  **For me to review the submission in depth I would expect at the very
least a package that passes CRAN QC and also includes a _runnable_
vignette that showcases the package.**

Thanks. The software has now been published to CRAN: <https://cran.r-project.org/web/packages/geodl/index.html>

We have also built a website for the package with associated vignettes and references: <https://wvview.org/geodl/index.html> **Also, the package should provide step-by-step instructions so that
someone like me [who is not familiar with the author's development
environment] can install the package, download a few datasets, and run
a few illustrative tests. As it stands, the package is unusable and
does not fulfil the laudable aims set out in the introduction.**

We have now provided links in the text to aid in installation. Since the most difficult component is installing torch and CUDA, we have provided a link to the official installation instructions associated with the torch package: <https://torch.mlverse.org/docs/articles/installation>.

We have also built a website for the package with associated vignettes and references: <https://wvview.org/geodl/index.html>

A total of ten vignettes were built.

Associated text:

*The geodl package is available on CRAN (*[*https://cran.r-project.org/web/packages/geodl/index.html*](https://cran.r-project.org/web/packages/geodl/index.html)*) and can be installed in R using install.packages(). The source code is available on GitHub (*[*https://github.com/maxwell-geospatial/geodl*](https://github.com/maxwell-geospatial/geodl)*), and the package can alternatively be installed from GitHub using the remotes package [46]. Please consult the following page for details on how to install torch and its dependencies:* [*https://torch.mlverse.org/docs/articles/installation*](https://torch.mlverse.org/docs/articles/installation)*. This page also links to documentation for configuring the environment for GPU-based computation, which is necessary to train CNN-based semantic segmentation models. The package’s official website is available at* [*https://wvview.org/geodl/index.html*](https://wvview.org/geodl/index.html)*. The package webpage complements the material presented here by providing code and detailed vignettes demonstrating the package’s workflow and associated functions. The data required to implement the examples have been provided via FigShare [47]:* [*https://figshare.com/articles/dataset/geodl_example_data/23835165*](https://figshare.com/articles/dataset/geodl_example_data/23835165)*. A separate and smaller version of the data without the image chips used in the training workflow examples is also available on FigShare [48]:* [*https://figshare.com/articles/dataset/geodl_example_data_no_chips_/26824909*](https://figshare.com/articles/dataset/geodl_example_data_no_chips_/26824909)*. More general torch examples are provided on the torch website:* [*https://torch.mlverse.org/*](https://torch.mlverse.org/)*.*

**Quite often I read things like "R package XXX has been developed..."
and it is not clear whether these have been developed by the author or
someone else. Clarify this in the writing.**

Thanks. We have tried to be clearer throughout the text and within the figures and tables as to the source of the different functions and tools. The primary sources of confusion seem to be with the terra and luz packages, so we have tried to be especially careful when discussing these packages. Please see the edited text.

**REVIEWER 2**

**Reviewer #2: General Comments

The manuscript describes the functionality and use of an open-source R package in some detail, with both the package and supplementary data readily available. It argues that the major benefits of the geodl package are that it replicates the functionality of proprietary software (mentioning ArcGIS Pro in particular), as well as being a 'base R' implementation. The accompanying diagrams and example of usage provided help to elucidate the process behind the methods in the package, as well as give an intuitive idea of how the package functionality is structured. I would recommend this manuscript for publication, subject to my suggestions below.**

Thanks for reviewing our manuscript. **As the primary goal is to make established methods more accessible, there is no real demand (in my eyes) for there to be methodological development in this work. However, I feel the argument of the manuscript would be improved by a comparison of geodl's results with existing methods (commercial or otherwise), where such comparisons can be made. Two main metrics for comparison that immediately come to mind are computational efficiency and quality of result; it would be particularly interesting to see the difference between using this base R implementation and a Python-in-R implementation (I presume using, eg. reticulate). Comparative limitations do not preclude this package having use, but should be addressed where they exist.**

Our primary goal for publishing a paper alongside our package and associated documentation was to provide a detailed articulation of the packages designs to aid users in citing it and explaining its implementation in their own studies. We have tried to be clearer about this purpose upfront. We have ended the needs and justification section with a statement of the goals of the paper:

*This article has two primary purposes. First, it provides an introduction to the geospatial semantic segmentation workflow implemented by geodl and introduces reference materials and documentation to help researchers and analysts implement the package for their own needs. Second, it provides a detailed documentation of the package’s functions and design philosophy, including the configuration of the provided UNet architectures and their associated modules and the implementation and parameterization of the provided loss and assessment metrics. For specific code-based examples, please see the package webpage and associated vignettes.*

We feel that there is particular value in providing a detailed discussion of the implementation of the UNet architectures and associated modules, loss functions, and assessment metrics. We often struggle with the lack of clarity in implementations when using other software packages, so our goal here is to alleviate such issues for this package to aid researchers.

We would like to refrain from providing comparisons to other implementations in regard to speed and model performance. There are many factors that could impact this, such as system hardware and the specific mapping or modeling problem. Further, the speed of the implementation is more dependent on the torch package and associated C++ backend than our own implementation.

We have expanded our discussion of existing implementations in the “Need and justification” section to more clearly frame the packages need and highlight the lack of R-based implementations and that R is lagging behind Python, especially when considering that most deep learning implementations in R act as wrappers to Python implementations. In short, we frame the need for the package in the context of (1) lack of deep learning tools in R, (2) expanding the geospatial tools available in R, (3) using the functionality of terra in R to support deep learning, (4) simplifying the software stack by not requiring the installation of a Python environment, and (5) growing an ecosystem around the terra package. We hope this clearly articulates our goals for this manuscript and package.

*Open-source software, data science tools, and datasets have had a positive impact on how science is conducted, and the development of new tools and techniques has hastened the speed of scientific innovation and the transition of knowledge to action while also fostering reproducible and transparent research [13,14]. Open-source software and datasets, application programming interfaces (APIs), modules, and code libraries have reduced cost, increased the accessibility of research tools, and become key components of research and training infrastructure. Open-source DL tools (i.e., code to support data preparation; model creation, training, and validation; and inference to new data) are currently well developed in the Python [15] language and data science environment, resulting from the development of libraries including Tensorflow [16], Keras [17], and PyTorch [18]. This can be partially attributed to the development of DL amongst the computer vision research community, wherein Python is more popular than other common data science environments [19]. Base DL libraries, such as Tensorflow and PyTorch, have been extended with additional libraries specifically focused on semantic segmentation tasks. These libraries include Kornia [20], MMSegmentation [21], PixelLib [22], and Segmentation Models [23,24]. The TorchGeo [25] library supports the use of geospatial data within the PyTorch ecosystem.*

*Many data, geospatial, and Earth scientists use R [26], which was originally developed for statistical computing, data wrangling, and data analysis. This flexible environment offers a large number of specialized packages, familiarity and ease of use, quality of documentation, a large user base, and available integrated development environments (IDEs), such as RStudio [28]. Specialized R packages include those targeted for machine learning tasks such as clustering (e.g., dtwclust [29]) as well as those used for data wrangling (e.g., tidyverse [30]).*

*A large set of R packages have already been developed for reading, working with, and analyzing geospatial data specifically, such as sf [31], terra [32], stars [33], and tmap [34]. The recent release of the terra package, which replaced the raster package [35] and currently (in early 2024) has had over six million downloads from the Comprehensive R Archive Network (CRAN) since its release in March 2020 based on download statistics obtained using the dlstats package [36], has improved computational efficiency for processing large raster grids, including digital elevation data and multispectral imagery [32]. Using the C++ language [37] via the rcpp package [38,39], terra allows for reading in portions of large raster grids from disk as opposed to reading the entire dataset to memory, which has greatly improved the practical application of raster-based geospatial data handling and analysis in R [32].*

*Many DL tools in R rely on Python and act as a wrapper for Python libraries. Using the reticulate package [40], R packages such as keras [41], tensorflow [42], and fastai [43] allow for the execution of Python-based DL from the R environment using R code, and therefore requires the installation of Python environments and libraries. Highlighting the interest in implementing DL in R, the keras package has had over two million downloads from CRAN since its release in July 2017. The recently released torch package [44], which is written in R and C++ and built directly on libtorch (the PyTorch C++ backend) [45], simplifies the software stack by eliminating the need for the Python “middleman”, thus avoiding reliance on Python and the associated issues stemming from incorrect versions of software or libraries and complications in setting up analytical environments, as well as the difficulties with troubleshooting errors. This is a large step forward in developing a DL experimentation environment and ecosystem native to R and C++. The torch package has been downloaded from CRAN over 160,000 times since its release in August 2020. We argue that there will be increased use of torch as an ecosystem of packages develops around it, as has occurred for the Python-based PyTorch implementation.*

**The manuscript is well-written, on the whole, and structured logically. It would be useful to see more reflective commentary on some of the choices made. In particular:
- The choice of an 'opinionated' stance on data dimensionality is reasonable from the point of view of code stability but, in my experience, can conflict with user accessibility, especially where a user is more familiar with a different data representation. A discussion on the rationale behind the choices made would help;**

Thanks. We further elaborated on this point in the revised manuscript.

1. *When using the implemented models, loss metrics, and/or inference tools, data must be provided with the following required shapes and data types. All predictor variable tensors are expected to have a shape of [Channels/Predictors, Width, Height], even if only one predictor variable is provided, and to have a 32-bit float data type. All targets or labels are expected to have a shape of [Class Indices, Width, Height] and a long integer data type. Our defineSegDataSet() function processes input chips to meet these criteria and can accept a variety of raster-based input data; as a result, it is not difficult to adhere to the package standards.*

**- How does the removal of incomplete chips affect the final inference? Are there any possible future developments to, e.g., impute from non-rectangular chips, and how would this inclusion affect the resulting output?**

Thanks. We added the following sentence. We hope this addresses your concern.

*If a chip is incomplete or has null values, generally resulting from a non-rectangular raster extent, that chip will not be written to disk; only complete chips are produced. In future releases, we plan to add additional functionality for handling non-rectangular extents.*

**- Does the availability of only three activation functions limit the applicability of the package, or is there an argument that can be made to explain why these are sufficient for all applications?**

The ReLU activation function is commonly used throughout UNet architectures. Thus, providing only three activation functions is not limiting. Adding options to use leaky ReLU or swish thus expands upon what is generally available when using UNet. Note that other activations are used in the context of loss and assessment metric calculations, such as softmax; however, this transformation is applied outside of the model. In other words, the model returns raw logits that can be converted to probabilities for loss and assessment metric calculations as needed. This is common practice in designing deep learning architectures. We hope this addresses your concern.  **Finally, it would be helpful to standardise the referencing provided within the manuscript, which oscillates between "statement from Foo et al. (2010)" to "statement [21]" to "statement from Foo et al. [21]". Where possible, references to software packages should reference publications, rather than the software itself: checking the R packages cited in particular, I found that Rcpp and psych both have publications that may be referenced, whose references can be found via** [**https://cran.r-project.org/web/packages//citation.html**](https://cran.r-project.org/web/packages/citation.html)**. Similarly, the landcover.ai dataset [44] has a non-arXiv link which can be found on the landcover.ai website.**

We have reviewed all citations in the package to update them for consistency. We use a Foo et al. [#] style as opposed to a Foo et al. (Year) style. We have used published papers as opposed to package documentation or arVix links where possible (For example, for Rcpp and the landcover.ai citation). We are willing to make additional changes as needed.  **Specific Comments: Manuscript
- pg 8: "The dataset consists of 123 United States (U.S.)..." Awkwardly worded: perhaps rephrase to "The dataset consists of 123 1:24,000 scale, 7.5-minute topographic maps from the United States, covering parts of Eastern Kentucky..."**

Thanks. This sentence was reworded to read as follows:

*“The dataset consists of 123 1:24,000-scale, 7.5-minute topographic maps from the United States covering parts of eastern Kentucky, 23 covering parts of eastern Ohio, and 25 covering parts of southwestern Virginia.”*

**- pg 19-20: Equation labelling inconsistent; arguably "if" and "else" should be non-italicised and justified left.**

Thanks. We have edited these equations as follows:

$ReLU= \left\{ \begin{aligned} activation if activation> 0 \\ 0 if activation \leq0 \end{aligned} \right.$ (Equation 1)

$Leaky ReLU= \left\{ \begin{aligned} activation if activation> 0 \\ activation*nst if activation \leq0 \end{aligned} \right.$ (Equation 2)

**- pg 25: "Dice score is actually and accuracy measure": should be *an* accuracy measure.**

This typo was correct. Thanks for catching this.

**- pg 26: Floating parentheses in equations: reformat these for clarity.**

The equations were updated. Thanks.

Multiclass Tversky loss = $\frac{1}{N}\sum_{j=1}^{C} (1-(\frac{\Sigma\hat{p}_{TP} + \varepsilon}{\Sigma\hat{p}_{TP}+\alpha\Sigma\hat{p}_{FN}+\beta\Sigma\hat{p}_{FP} +\varepsilon}))$ (Equation 5)

Modified Tversky Index (mTI) = $(\frac{\Sigma\hat{p}_{TP} + \varepsilon}{\Sigma\hat{p}_{TP}+\delta\Sigma\hat{p}_{FN}+(1-\delta)\Sigma\hat{p}_{FP} +\varepsilon})$ (Equation 9)

**- pg 27: "Values between 0.5 and 1 put more weight...": I would argue that this sentence is redundant, given the sentence above and the equations provided.**

Thanks. This sentence was removed.

**- pg 29: "however, user's can choose...": no apostrophe.**

Thanks. the apostrophe was removed.

**- pg 35: "probabilities of being applied of 0.5, 0.5, 0.1, and 0.2, respectively". This sentence is somewhat confusing, when combined with the statement that a maximum of one augmentation is performed on each chip. Do we mean that there is a 50% chance of a vertical flip, a 50% chance of a horizontal flip conditioned on no vertical flip, a 10% chance of a brightness adjustment conditioned on no flipping whatsoever, etc? Or should these probabilities sum to 1, and every chip receives exactly one augmentation?**

Thanks. We tried to clarify this:

*For the training set, a maximum of one augmentation was performed per chip, either a vertical or horizontal flip with a 0.5 probability of being applied. In other words, no more than one augmentation was applied to a single chip, and there was a 50% chance of applying the single augmentation. If no augmentation is applied, the original chip is returned.*

**Code Comments
The package installed with no complications using devtools::install_github(). Replication scripts are easy to parse and suitably commented. Where code has been used to generate a manuscript figure, it would be advisable to indicate that in the relevant script file (a comment along the lines of ## FIGURE GENERATION CODE ## would suffice).**

Thanks. The software has now been published to CRAN: <https://cran.r-project.org/web/packages/geodl/index.html>

We have also built a website for the package with associated vignettes and references: <https://wvview.org/geodl/index.html> **I would advise making replication data easier to access, either by directly referencing the source and modifying the replication scripts to account for this, or by splitting the geodldata.zip file into sections so that specific examples can be checked without downloading a 15Gb file. I understand the difficulty of doing so given the application, but as a general rule more lightweight data examples would encourage engagement with the package for first-time readers and users.**

The main size issue here arose from the inclusion of large sets of image chips to train and assess models. We have now provided a second version of the data on FigShare: one that includes the image chips and one that does not. The vignettes are now available on CRAN and also on the package website with rendered output.

We have added a paragraph to explain the sources of data and documentation in the “Need and justification” section:

*The geodl package is available on CRAN (*[*https://cran.r-project.org/web/packages/geodl/index.html*](https://cran.r-project.org/web/packages/geodl/index.html)*) and can be installed in R using install.packages(). The source code is available on GitHub (*[*https://github.com/maxwell-geospatial/geodl*](https://github.com/maxwell-geospatial/geodl)*), and the package can alternatively be installed from GitHub using the remotes package [46]. Please consult the following page for details on how to install torch and its dependencies:* [*https://torch.mlverse.org/docs/articles/installation*](https://torch.mlverse.org/docs/articles/installation)*. This page also links to documentation for configuring the environment for GPU-based computation, which is necessary to train CNN-based semantic segmentation models. The package’s official website is available at* [*https://wvview.org/geodl/index.html*](https://wvview.org/geodl/index.html)*. The package webpage complements the material presented here by providing code and detailed vignettes demonstrating the package’s workflow and associated functions. The data required to implement the examples have been provided via FigShare [47]:* [*https://figshare.com/articles/dataset/geodl_example_data/23835165*](https://figshare.com/articles/dataset/geodl_example_data/23835165)*. A separate and smaller version of the data without the image chips used in the training workflow examples is also available on FigShare [48]:* [*https://figshare.com/articles/dataset/geodl_example_data_no_chips_/26824909*](https://figshare.com/articles/dataset/geodl_example_data_no_chips_/26824909)*. More general torch examples are provided on the torch website:* [*https://torch.mlverse.org/*](https://torch.mlverse.org/)*.*

**For function documentation in R, the first line of the documentation should include a (short) description of the function usage - looking at help(package = "geodl") in comparison with, eg, help(package = "dplyr") should make clear why.**

The package documentation was edited as required during the CRAN submission process. Thanks. **I would also like to wish the authors luck in submitting their package to CRAN, which can be a gruelling and occasionally dispiriting process, but well worth the time spent in doing so.**

Thanks. The software has now been published to CRAN: <https://cran.r-project.org/web/packages/geodl/index.html>

The process was stressful, but it did improve the package and associated documentation. Thanks.

**REVIEWER 3**

**Many thanks for the invitation to review this paper. I have reviewed the manuscript as well as the coded examples on figshare, as linked in the paper. For full disclosure, I am a specialist in deep learning methods, but I am not an expert in spatial methods, and so my review is focused more generally on the presentation of a software contribution.**

Thanks for reviewing our paper.  **Overall, there is little doubt that this manuscript demonstrates a considerable amount of work to develop a near end-to-end package for conducting spatial analysis using DL-based architectures in R. It builds on industry-standard libraries, has sensible naming conventions that will help encourage its adoption, and installs cleanly on the latest version of R (4.4.1). That said, as a research paper, I think the manuscript needs to signal a more coherent contribution that goes beyond describing the various constituent functions and**

**methods.**

Thanks for this comment.
 **\## Comments:

1. The paper is not currently accepted on CRAN — I do not recommend proceeding with this manuscript until the repository has accepted this package.**

Thanks. The software has now been published to CRAN: <https://cran.r-project.org/web/packages/geodl/index.html>

We have also built a website for the package with associated vignettes and references: <https://wvview.org/geodl/index.html> **2. The central contention of the paper is that, at present, state-of-the-art DL methods for spatial analyses are unavailable in R, without the need for cumbersome backends to Python. I fully agree that using the C++ backend is a welcome departure from Python-routed alternatives. My concern, however, is that the sort of user implementing these methods will either a) be fluent in a more conventional DL language such that they wouldn’t consider running it in R in the first place, or b) would be less familiar with DL in general, and thus find the package and pipeline intimidating. I would expect an original article to try and engage with these two archetypal users more specifically.**

Thanks for this comment. We have tried to more clearly articulate the purpose of the article in the “Need and justification” section:

*This article has two primary purposes. First, it provides an introduction to the geospatial semantic segmentation workflow implemented by geodl and introduces reference materials and documentation to help researchers and analysts implement the package for their own needs. Second, it provides a detailed documentation of the package’s functions and design philosophy, including the configuration of the provided UNet architectures and their associated modules and the implementation and parameterization of the provided loss and assessment metrics. For specific code-based examples, please see the package webpage and associated vignettes.*

We have now broken our discussion into two broad components: “Overview of the geodl workflow: and “Details of package implementation”.

The first component is meant for users that are less interested in the implementation and are more interested in the process/workflow. We have included a new figure (Figure 1) that attempts to represent the workflow in as simple terms as possible:

We have also included a new table, Table 1, that summarizes key deep learning terminology:

| **Term** | **Explanation** |
| --- | --- |
| Chip | Image or raster-based predictor variable extent of a defined size (e.g., 64×64, 128×128, 256×256, or 512×512 cells) used as samples to train and validate models. |
| Mask or label | Raster-based reference data associated with each chip in which each cell is assigned an integer class label. |
| Training data | Data partition of chips and associated masks used to guide the parameter updates during the training process. |
| Validation data | Data partition of chips and associated masks which are commonly predicted at the end of each training epoch to assess for model generalization. |
| Test data | Withheld data partition of chips and associated masks used to assess the final model. |
| Loss metric | Measure used to guide parameter updates during the training process. The goal of the training process is to minimize this metric. Examples include cross entropy loss, Dice loss, and Tversky loss. |
| Assessment metric | Measure of model performance that can be monitored during the training process or calculated from the withheld test data. These metrics are not used to guide the parameter updates. Examples include overall accuracy, F1-score, recall, and precision. |
| Dataset | A torch class used to define how individual samples are processed before being provided to the dataloader and model. |
| Dataloader | A torch class that defines how mini-batches of data are provided to the model during training and inference. |
| Mini-batch | A subset of samples that are collectively provided to the model using a dataloader. Commonly, parameter updates are made after each mini-batch is processed as opposed to after one complete iteration over all training samples. |
| Epoch | One iteration over the entire training set or all mini-batches making up the entire training dataset. |
| Training loop | Process of iteratively training the model using the training data, a loss metric, backpropagation, and an optimization algorithm. |
| Optimizer | Algorithm used to update the model’s trainable parameters. Examples include mini-batch stochastic gradient descent, RMSProp, and Adam. |
| Hyperparameter | A model or training process setting that is not learned during the training process. Examples include the optimizer’s learning rate and the number of feature maps generated by each block in the encoder. |
| Parameter | A model component that is updated during the training process, such as weights and biases for convolutional kernels and gain and shift for batch normalization. |

We have now published vignettes/articles on the package website (<https://wvview.org/geodl/index.html>) and on CRAN (<https://cran.r-project.org/web/packages/geodl/index.html>) to demo all components of the package. A total of ten vignettes were generated. Two vignettes provide example implementations of the entire workflow (one for a multiclass classification and one for a binary classification). The other vignettes cover specific components, such as creating raster masks, creating image chips, configuring the loss function, assessing model output, and making predictions to spatial data.

In the “Needs and justification” section, we explain and highlight the package resources:

*The geodl package is available on CRAN (**<https://cran.r-project.org/web/packages/geodl/index.html>) and can be installed in R using install.packages(). The source code is available on GitHub (*[*https://github.com/maxwell-geospatial/geodl*](https://github.com/maxwell-geospatial/geodl)*), and the package can alternatively be installed from GitHub using the remotes package [46]. Please consult the following page for details on how to install torch and its dependencies:* [*https://torch.mlverse.org/docs/articles/installation*](https://torch.mlverse.org/docs/articles/installation)*. This page also links to documentation for configuring the environment for GPU-based computation, which is necessary to train CNN-based semantic segmentation models. The package’s official website is available at* *<https://wvview.org/geodl/index.html>. The package webpage complements the material presented here by providing code and detailed vignettes demonstrating the package’s workflow and associated functions. The data required to implement the examples have been provided via FigShare [47]:* [*https://figshare.com/articles/dataset/geodl_example_data/23835165*](https://figshare.com/articles/dataset/geodl_example_data/23835165)*. A separate and smaller version of the data without the image chips used in the training workflow examples is also available on FigShare [48]:* [*https://figshare.com/articles/dataset/geodl_example_data_no_chips_/26824909*](https://figshare.com/articles/dataset/geodl_example_data_no_chips_/26824909)*. More general torch examples are provided on the torch website:* [*https://torch.mlverse.org/*](https://torch.mlverse.org/)*.*

The “Details of package implementation” section explains the package in detail. We feel that there is particular value in providing a detailed discussion of the implementation of the UNet architectures and associated modules, loss functions, and assessment metrics. We often struggle with the lack of clarity in implementations when using other software packages, so our goal here is to alleviate such issues for this package to aid researchers. In other words, having access to a peer reviewed article that explains the implementation in detail is of value when the package will be used in a research context.  **3. Put another way, I am not sure who this paper is for, and therefore it is hard to judge how useful it is. The description of methods relies on a reasonably proficient understanding of DL methods. For example, the paper defers the entire training loop to another package, luz, which requires the reader *and* implementer to understand DL to a reasonable degree (even if luz is high-level enough to remove some of the coding pain). And if the reader has that knowledge, are they likely to be solely working in R? So, at present, the paper does not do enough to convince me that the sort of analyst this paper targets would want to adopt this package.**

As noted for your prior comments, we have tried to be clearer regarding the purpose of the manuscript. The “Overview of geodl workflow” section is meant for a non-technical user. This section is also supported by vignettes/articles provided on the package website and on CRAN. These resources are introduced early in the article. We feel that users of R geospatial packages (e.g., terra, sf, tmap, and leaflet) will have interest in being able to use R for deep learning. We also feel that the provided vignettes, which are very descriptive and include explanatory text, will be of value to users that are new to deep learning.

The “Details of package implementation” section provides a detailed discussion of the package implementation. We feel strongly that offering a detailed discussion of the implementation is important to aid authors that are using the tool in a research context.

In short, we hope that our edits and additions make the purpose of the manuscript clearer. Also, we feel that the published vignettes and website will be of great value to those less familiar with deep learning. We argue that the manuscript and associated resources (vignettes and website) collectively service a variety of users.  **4. I think the real value of a software paper is providing indicative simulations and analysis that helps interested users navigate the software and pipeline. The paper does a good job of describing functions, but could focus more on the consequences of these implementations. For more technical users, for example, what do we gain by not off-loading our training to a separate coding language? Can you provide benchmarks that show that users are not sacrificing compute speed/memory efficiency, compared to similar analysis in Python? And/or can you demonstrate that the *full* analysis pipeline would be more convoluted without geodl because, in this area, the leading analytical packages reside in R rather than other languages?**

As we have edited the paper, we have tried to highlight the value of (1) being able to implement a workflow in a single language, (2) make use of other R packages, such as the tidyverse packages, during the workflow, and (3) the value of expanding the use of R in a deep learning context and building on the torch package. We have also tried to highlight that terra is an excellent package for handling geospatial raster data in R and that the existence of terra and torch presents an opportunity to expand geospatial deep learning in R. Through our generated vignettes/articles we demonstrate complete workflows and also key components of the process with associated explanations.

We would like to refrain from providing comparisons to other implementations in regard to speed and model performance. There are many factors that could impact this, such as system hardware and the specific mapping or modeling problem. Further, the speed of the implementation is more dependent on the torch package and associated C++ backend than our own implementation. We are not arguing that this implementation is faster than those based on Python and/or R+Python. Instead, we argue that the use of torch and terra presents an opportunity to expand the geospatial deep learning capabilities of R without the need for a Python environment and that not having to create, manage, and update Python environments can greatly simplify the implementation of deep learning for R users. We hope this addresses your comment.

**5. I did not find the design philosophy section convincing. This discussion was more about constraints on the input data, rather than an overview of the method itself. Figure 1 helps, but is really complicated and, I suspect, could be off-putting to potential users. I would have expected a clearer, high-level, and narrative description of the pipeline itself that would then foreground the rather technical description of the constituent functions in the following sections.**

Thanks for this comment. We have now offered a high-level narrative as a new section: “Overview of geodl workflow”. We hope this addresses your concerns.

We have included a new figure (Figure 1) that attempts to represent the workflow in as simple terms as possible.

**6. The manuscript could also provide a more comprehensive walkthrough of the pipeline *in the paper*. I like the two applied examples, and think this portion of the paper could be built out more. Although the code shared on the Github repository and figshare is clear and well-annotated, it would be important to have at least one comprehensive demonstration of the coding pipeline, which is discussed in depth. At the moment the description of the implementation is fine, but a) there is no code in the text to follow along with, and b) the discussion of hyperparameters is descriptive rather than explanatory (e.g. “a mini-batch size of 15 was used and the model was trained for ten epochs”, but why were these chosen in this context?) I also think less time could be spent analysing the performance metrics of the trained models (since, as I understood the paper, the DL architectures are not novel) and more time spent *demonstrating* how changes to model parameters etc. influence the outputs of the model.**

We have opted to provide code-based walkthroughs in the vignettes/articles that are now available on the package website and on CRAN. The vignettes include both the code and explanations. In the paper, we have tried to focus on the implementation details. We made this decision based on the following:

1. The code will likely need to be updated on a regular basis as the package is updated and/or the dependencies (torch, terra, and luz) are updated. This is easier to implement within vignettes/articles as opposed to in a published paper.
2. We hope to continue to add vignettes/articles as additional functionality is provided or to highlight specific issues or use cases (such as using sparsely labeled masks). Thus, the examples are less static when implemented as vignettes/articles as opposed to within the text of the paper.
3. The package website is linked to the GitHub source code, so users can more easily report issues or make suggestions.
4. We wanted the primary goal of the paper to be presenting a detailed discussion of the package to support users and researchers while providing examples via the package webpage.

**## Minor things:**

**7. The package seems to have limited backwards compatibility due to its dependencies (its install failed on R 4.3 because it could not install luz/terra/etc.)**

Thanks for this comment. Since the package is built to extend the use of torch, terra, and luz, we are, unfortunately, tied to these constraints. However, we argue that the package is of use even with limited backwards compatibility.

**8. I would shift the discussion of datasets from pp.8-9 to the beginning of the applied examples section later in the paper**

Thanks for this comment. We have restructured the paper. We are willing to move this content to a different location in the edited manuscript if you still feel that it should be relocated. Thanks.

**REVIEWER 4**

**The authors present the geodl software package to facilitate deep learning-based pixel-level classification of images in R (with emphasis on geospatial data). It builds on the functionality of key R packages for deep learning (torch, luz) and geospatial data analysis (terra, sf), among others; the authors demonstrate a deep understanding of the R ecosystem and geodl integrates nicely with related packages. geodl helps fulfill a need for more deep learning tools within R, especially as geospatial practitioners commonly use R. The methodology and model architecture are sound and follow established deep learning principles. The package is well-designed, with sensible defaults and a good range of options for customizing the model, fitting process, and loss functions. The package itself is well-documented and available on Github. The modeling and package details are presented clearly in the paper and are easy-to-follow, with a good amount of background so that the various parameters and options are not overwhelming. Two example datasets (one binary and one multiclass) are presented as case studies to illustrate package usage.**

Thanks for reviewing our manuscript. **Minor comments for improvement:

- The case studies are somewhat short on detail relative to the earlier sections. For example, are package defaults used (other than the settings explicitly mentioned), and how might we know if they are appropriate? What is the hardware and training time required for these case studies?**

Thanks. We have now provided more detailed examples as separate vignettes/articles on the package webpage. We opted to do this as opposed to expand the examples in the paper so as not to further lengthen the paper and also because the examples can be more regularly updated or expanded if provided on the package website as opposed to within the text of the paper.

Thanks.

The software has now been published to CRAN: <https://cran.r-project.org/web/packages/geodl/index.html>

We have also built a website for the package with associated vignettes and references: <https://wvview.org/geodl/index.html>

**- Equations (5) and (9) appear to have some typos.**

Thanks for catching this. We have updated the equation as follows. We believe these are now correct.

Multiclass Tversky loss = $\frac{1}{N}\sum_{j=1}^{C} (1-(\frac{\Sigma\hat{p}_{TP} + \varepsilon}{\Sigma\hat{p}_{TP}+\alpha\Sigma\hat{p}_{FN}+\beta\Sigma\hat{p}_{FP} +\varepsilon}))$ (Equation 5)

Modified Tversky Index (mTI) = $(\frac{\Sigma\hat{p}_{TP} + \varepsilon}{\Sigma\hat{p}_{TP}+\delta\Sigma\hat{p}_{FN}+(1-\delta)\Sigma\hat{p}_{FP} +\varepsilon})$ (Equation 9)

**REVIEWER 5**

**Making complex computational tools easier to use by developing streamlined workflows addressing particular needs is a valuable goal. The geodl package aims to perform that task by facilitating deep learning of spatial data in R.**

Thanks for reviewing our manuscript.  **This could be a valuable contribution, but it isn't clear who the audience is, and the manuscript could benefit from substantial restructuring once that question is decided. I come to this as a fairly naive user of DL but a very experienced user of R, and was excited to see a manuscript and package that addressed my situation. Even the abstract was mixed in intent: it began with an overview of DL and spatial data, a couple of sentences on why a new R package was desirable, and then disappeared into DL jargon with no explanation.**

Thanks for this comment. We have tried to more clearly articulate the purpose of the article in the “Need and justification” section:

*This article has two primary purposes. First, it provides an introduction to the geospatial semantic segmentation workflow implemented by geodl and introduces reference materials and documentation to help researchers and analysts implement the package for their own needs. Second, it provides a detailed documentation of the package’s functions and design philosophy, including the configuration of the provided UNet architectures and their associated modules and the implementation and parameterization of the provided loss and assessment metrics. For specific code-based examples, please see the package webpage and associated vignettes.*

We have broken our discussion into two broad components: “Overview of the geodl workflow” and “Details of package implementation”.

The first component is meant for users that are less interested in the implementation and are more interested in the process/workflow. We have included a new figure (Figure 1) that attempts to represent the workflow in as simple terms as possible:

We have also included a new table, Table 1, that summarizes key deep learning terminology:

| **Term** | **Explanation** |
| --- | --- |
| Chip | Image or raster-based predictor variable extent of a defined size (e.g., 64×64, 128×128, 256×256, or 512×512 cells) used as samples to train and validate models. |
| Mask or label | Raster-based reference data associated with each chip in which each cell is assigned an integer class label. |
| Training data | Data partition of chips and associated masks used to guide the parameter updates during the training process. |
| Validation data | Data partition of chips and associated masks which are commonly predicted at the end of each training epoch to assess for model generalization. |
| Test data | Withheld data partition of chips and associated masks used to assess the final model. |
| Loss metric | Measure used to guide parameter updates during the training process. The goal of the training process is to minimize this metric. Examples include cross entropy loss, Dice loss, and Tversky loss. |
| Assessment metric | Measure of model performance that can be monitored during the training process or calculated from the withheld test data. These metrics are not used to guide the parameter updates. Examples include overall accuracy, F1-score, recall, and precision. |
| Dataset | A torch class used to define how individual samples are processed before being provided to the dataloader and model. |
| Dataloader | A torch class that defines how mini-batches of data are provided to the model during training and inference. |
| Mini-batch | A subset of samples that are collectively provided to the model using a dataloader. Commonly, parameter updates are made after each mini-batch is processed as opposed to after one complete iteration over all training samples. |
| Epoch | One iteration over the entire training set or all mini-batches making up the entire training dataset. |
| Training loop | Process of iteratively training the model using the training data, a loss metric, backpropagation, and an optimization algorithm. |
| Optimizer | Algorithm used to update the model’s trainable parameters. Examples include mini-batch stochastic gradient descent, RMSProp, and Adam. |
| Hyperparameter | A model or training process setting that is not learned during the training process. Examples include the optimizer’s learning rate and the number of feature maps generated by each block in the encoder. |
| Parameter | A model component that is updated during the training process, such as weights and biases for convolutional kernels and gain and shift for batch normalization. |

We have now published vignettes/articles on the package website (<https://wvview.org/geodl/index.html>) and on CRAN (<https://cran.r-project.org/web/packages/geodl/index.html>) to demo all components of the package. A total of ten vignettes were generated. Two vignettes provide example implementations of the entire workflow (one for a multiclass classification and one for a binary classification). The other vignettes cover specific components, such as creating raster masks, creating image chips, configuring the loss function, assessing model output, and making predictions to spatial data.

In the needs and justification section, we explain and highlight the package resources:

*The geodl package is available on CRAN (*[*https://cran.r-project.org/web/packages/geodl/index.html*](https://cran.r-project.org/web/packages/geodl/index.html)*) and can be installed in R using install.packages(). The source code is available on GitHub (*[*https://github.com/maxwell-geospatial/geodl*](https://github.com/maxwell-geospatial/geodl)*), and the package can alternatively be installed from GitHub using the remotes package [46]. Please consult the following page for details on how to install torch and its dependencies:* [*https://torch.mlverse.org/docs/articles/installation*](https://torch.mlverse.org/docs/articles/installation)*. This page also links to documentation for configuring the environment for GPU-based computation, which is necessary to train CNN-based semantic segmentation models. The package’s official website is available at* [*https://wvview.org/geodl/index.html*](https://wvview.org/geodl/index.html)*. The package webpage complements the material presented here by providing code and detailed vignettes demonstrating the package’s workflow and associated functions. The data required to implement the examples have been provided via FigShare [47]:* [*https://figshare.com/articles/dataset/geodl_example_data/23835165*](https://figshare.com/articles/dataset/geodl_example_data/23835165)*. A separate and smaller version of the data without the image chips used in the training workflow examples is also available on FigShare [48]:* [*https://figshare.com/articles/dataset/geodl_example_data_no_chips_/26824909*](https://figshare.com/articles/dataset/geodl_example_data_no_chips_/26824909)*. More general torch examples are provided on the torch website:* [*https://torch.mlverse.org/*](https://torch.mlverse.org/)*.*

The “Details of package implementation” section explains the package in detail. We feel that there is particular value in providing a detailed discussion of the implementation of the UNet architectures and associated modules, loss functions, and assessment metrics. We often struggle with the lack of clarity in implementations when using other software packages, so our goal here is to alleviate such issues for this package to aid researchers. In other words, having access to a peer reviewed article that explains the implementation in detail is of value when the package will be used in a research context.  **Is this manuscript primarily for:
- spatial scientists who might use DL more if it were easier to access in R? (me)
- experienced DL users who might use R more if it were easier to access DL?
- the general reader who might be encouraged to use both R and DL?**

Thanks for this comment. We hope that our answer to your prior comment addresses your concerns.  **Many parts of the manuscript are very technical on DL concepts and language, and thus more suited for the second class of users, but there isn't anything to my reading that would convince those data scientists to switch to R from their established (likely) PyTorch workflows.**

We feel strongly that there are many users that use the machine learning tools in R (such as caret and tidymodels) who would be interested in having access to deep learning tools in R as opposed to having to shift to another language for such analyses, or, as we have done in the past, use a hybrid workflow relying on both R and Python. We also feel that there is value in being able to set up a deep learning working environment that does not require maintaining an associated Python virtual environment. In short, we feel that there is a user base for this package. As we have edited the paper, we have tried to more clearly articulate the value of the package for end users. Specifically, we feel that the combination of torch and terra provides a unique opportunity to apply deep learning techniques to geospatial raster data in the R environment. We hope this addresses your concern.  **It seems to me that the first and third classes, less experienced DL practitioners who might have related skills and applications, would be the most useful target, especially for a general journal like this one. But the manuscript should then be refactored to better present the workflow, concepts, and techniques.**

We feel that the revised manuscript has been refactored to reflect your comments. As noted above, we have tried to divide the material into a discussion of the more general workflow (the “Overview of geodl workflow” section) and a section focused on the details of the implementation (“Details of package implementation”). We have also tried to more clearly explain our supplemental materials:

*The geodl package is available on CRAN (*[*https://cran.r-project.org/web/packages/geodl/index.html*](https://cran.r-project.org/web/packages/geodl/index.html)*) and can be installed in R using install.packages(). The source code is available on GitHub (*[*https://github.com/maxwell-geospatial/geodl*](https://github.com/maxwell-geospatial/geodl)*), and the package can alternatively be installed from GitHub using the remotes package [46]. Please consult the following page for details on how to install torch and its dependencies:* [*https://torch.mlverse.org/docs/articles/installation*](https://torch.mlverse.org/docs/articles/installation)*. This page also links to documentation for configuring the environment for GPU-based computation, which is necessary to train CNN-based semantic segmentation models. The package’s official website is available at* [*https://wvview.org/geodl/index.html*](https://wvview.org/geodl/index.html)*. The package webpage complements the material presented here by providing code and detailed vignettes demonstrating the package’s workflow and associated functions. The data required to implement the examples have been provided via FigShare [47]:* [*https://figshare.com/articles/dataset/geodl_example_data/23835165*](https://figshare.com/articles/dataset/geodl_example_data/23835165)*. A separate and smaller version of the data without the image chips used in the training workflow examples is also available on FigShare [48]:* [*https://figshare.com/articles/dataset/geodl_example_data_no_chips_/26824909*](https://figshare.com/articles/dataset/geodl_example_data_no_chips_/26824909)*. More general torch examples are provided on the torch website:* [*https://torch.mlverse.org/*](https://torch.mlverse.org/)*.*

*This article has two primary purposes. First, it provides an introduction to the geospatial semantic segmentation workflow implemented by geodl and introduces reference materials and documentation to help researchers and analysts implement the package for their own needs. Second, it provides a detailed documentation of the package’s functions and design philosophy, including the configuration of the provided UNet architectures and their associated modules and the implementation and parameterization of the provided loss and assessment metrics. For specific code-based examples, please see the package webpage and associated vignettes.*

Thanks. The software has now been published to CRAN: <https://cran.r-project.org/web/packages/geodl/index.html>

We have also built a website for the package with associated vignettes and references: <https://wvview.org/geodl/index.html>

**Currently it bounces from general to specific and back again; I might suggest that two vignettes be added to the geodl package, one for experienced DL users with all the technical details, and one integrating the various scripts into a cohesive workflow (actually, that's probably a couple of vignettes). Then the manuscript could concentrate more on why this package is necessary and useful, and demonstrating its use with a case study.**

We have generated a total of ten vignettes that are available on the package website and CRAN (links are provided above). One vignette demonstrates a complete workflow for a multiclass classification using the landcover.ai dataset while one demonstrates a binary classification using the topoDL dataset. The other eight vignettes cover the following topics: making spatial predictions using a trained model, making raster masks from vector geospatial data, creating image chips from raster-based predictor variables and associated masks, defining datasets and dataloaders for use in the training process, creating terrain derivatives from a digital terrain model, parameterizing the unified focal loss, using our implemented assessment metrics, and performing accuracy assessment using a dataloader, point locations, or raster grids.

We feel strongly that these supplemental vignettes effectively supplement the material presented in the paper, which is more focused on the design of the package.

We prefer to present code in the associated vignettes as opposed to in the manuscript so that they can be more easily updated. We also plan to add additional vignettes/articles in the future.

**Having a workflow diagram like Fig 1 that shows the functions from this and related packages is great. This should complement the workflow in examples/scripts. This is the kind of thing that I think would be most useful for this manuscript in a general journal. (Though I'm curious why you suggest QGIS instead of R for processing spatial data?)**

We have now provided two separate figures. Figure 1 offers a more general overview while Figure 2 offers a more detailed workflow. We have removed the statement regarding QGIS. We had included this before since R is not a great environment for drawing or digitizing vector geospatial data. If the user needed to generate vector-based reference data, QGIS is a good alternative to commercial software. However, this was not really necessary to state, so the comment was removed.

**Fig 1.** Overview of geodl workflow.

**Fig 2.** Conceptualization of geodl DL semantic segmentation workflow.

**Table 1: Is there a more meaningful ordering for these scripts than alphabetical, for instance with the data preparation earlier in the workflow?**

Note that Table 1 is now Table 2. We selected this ordering based on the importance to the package. The torch, terra, and luz packages are most important since the package is meant to extend and build on these packages. The torchvision package is also important since it is tightly integrated with torch and provides additional functionality for working with raster-based data. In geodl, it is key for implementing image augmentations. All other packages are more ancillary. In short, we feel that this is a logical order. However, we are willing to reorder the table if necessary.  **Table 2: At least one other package, yardstick, is used in the examples.**

We did not include yardstick in the table since it is not used internally within any of geodl’s functions. It is used in the examples/vignettes to compare the assessment metrics calculated by our functions with those calculated by yardstick. We apologize if we misunderstood your comment here.  **"Disturbance associated with surface coal mining is denoted on topographic maps with a brown or pink pattern."
This should be rephrased to indicate that you mean pink and brown in the signal processing sense.**

Thanks. We have updated the sentence to read as follows. We hope this addresses your concern.

*“Surface coal mining is denoted on topographic maps with a pink or brown pattern or symbology that is meant to represent surface disturbances.”* **"The DL workflow as implemented in open-source environments, such as PyTorch, is
complicated by inconsistent data representations, dimensionality, and/or data types due to different
developers using different conventions."
This and subsequent are worth emphasizing. This is a key reason a wrapper package like geodl can be so useful.**

Thanks. We feel that our edited “Need and justification” section highlights this. We are willing to make additional changes if necessary. **The manuscript describes some workflow, and then jumps into a technical description of UNet models. I don't understand all the abbreviations in Fig 4, and they don't seem to be explained. Here again, who is the audience? I'm not sure figs 4-9 and associated technical description are necessary.**

Please see our response to the next comment. We feel that there is value in explaining the details of the implementation for those that want to use it in research studies and need to be able to explain, in detail, the architecture of the models used. This is something we have struggled with when using other libraries/packages.

In the figures, we have now made sure that all abbreviations have been defined in the figure legend or the figure caption. When necessary, we have also directed readers to Table 3 (which was Table 2 in the original version) that explains the function parameter names.

We have tried to better articulate the purpose of the article and provide clear links to package resources.  **Overall, I would find this to be a more useful contribution if it focused more on the application of this package, in general (philosophy, implementation) and case studies. I'm clearly coming from a certain perspective, but one that represents a broad readership.**

Thanks for this comment. We have tried to more clearly articulate the purpose of the article in the needs and justification section:

*This article has two primary purposes. First, it provides an introduction to the geospatial semantic segmentation workflow implemented by geodl and introduces reference materials and documentation to help researchers and analysts implement the package for their own needs. Second, it provides a detailed documentation of the package’s functions and design philosophy, including the configuration of the provided UNet architectures and their associated modules and the implementation and parameterization of the provided loss and assessment metrics. For specific code-based examples, please see the package webpage and associated vignettes.*

We have broken our discussion into two broad components: “Overview of the geodl workflow” and “Details of package implementation”.

The first component is meant for users that are less interested in the implementation and are more interested in the process/workflow. We have included a new figure (Figure 1) that attempts to represent the workflow in as simple terms as possible:

We have also included a new table, Table 1, that summarizes key deep learning terminology:

| **Term** | **Explanation** |
| --- | --- |
| Chip | Image or raster-based predictor variable extent of a defined size (e.g., 64×64, 128×128, 256×256, or 512×512 cells) used as samples to train and validate models. |
| Mask or label | Raster-based reference data associated with each chip in which each cell is assigned an integer class label. |
| Training data | Data partition of chips and associated masks used to guide the parameter updates during the training process. |
| Validation data | Data partition of chips and associated masks which are commonly predicted at the end of each training epoch to assess for model generalization. |
| Test data | Withheld data partition of chips and associated masks used to assess the final model. |
| Loss metric | Measure used to guide parameter updates during the training process. The goal of the training process is to minimize this metric. Examples include cross entropy loss, Dice loss, and Tversky loss. |
| Assessment metric | Measure of model performance that can be monitored during the training process or calculated from the withheld test data. These metrics are not used to guide the parameter updates. Examples include overall accuracy, F1-score, recall, and precision. |
| Dataset | A torch class used to define how individual samples are processed before being provided to the dataloader and model. |
| Dataloader | A torch class that defines how mini-batches of data are provided to the model during training and inference. |
| Mini-batch | A subset of samples that are collectively provided to the model using a dataloader. Commonly, parameter updates are made after each mini-batch is processed as opposed to after one complete iteration over all training samples. |
| Epoch | One iteration over the entire training set or all mini-batches making up the entire training dataset. |
| Training loop | Process of iteratively training the model using the training data, a loss metric, backpropagation, and an optimization algorithm. |
| Optimizer | Algorithm used to update the model’s trainable parameters. Examples include mini-batch stochastic gradient descent, RMSProp, and Adam. |
| Hyperparameter | A model or training process setting that is not learned during the training process. Examples include the optimizer’s learning rate and the number of feature maps generated by each block in the encoder. |
| Parameter | A model component that is updated during the training process, such as weights and biases for convolutional kernels and gain and shift for batch normalization. |

We have now published vignettes/articles on the package website (<https://wvview.org/geodl/index.html>) and on CRAN (<https://cran.r-project.org/web/packages/geodl/index.html>) to demo all components of the package. A total of ten vignettes were generated. Two vignettes provide example implementations of the entire workflow (one for a multiclass classification and one for a binary classification). The other vignettes cover specific components, such as creating raster masks, creating image chips, configuring the loss function, assessing model output, and making predictions to spatial data.

In the “Need and justification” section, we explain and highlight the package resources:

*The geodl package is available on CRAN (*[*https://cran.r-project.org/web/packages/geodl/index.html*](https://cran.r-project.org/web/packages/geodl/index.html)*) and can be installed in R using install.packages(). The source code is available on GitHub (*[*https://github.com/maxwell-geospatial/geodl*](https://github.com/maxwell-geospatial/geodl)*), and the package can alternatively be installed from GitHub using the remotes package [46]. Please consult the following page for details on how to install torch and its dependencies:* [*https://torch.mlverse.org/docs/articles/installation*](https://torch.mlverse.org/docs/articles/installation)*. This page also links to documentation for configuring the environment for GPU-based computation, which is necessary to train CNN-based semantic segmentation models. The package’s official website is available at* [*https://wvview.org/geodl/index.html*](https://wvview.org/geodl/index.html)*. The package webpage complements the material presented here by providing code and detailed vignettes demonstrating the package’s workflow and associated functions. The data required to implement the examples have been provided via FigShare [47]:* [*https://figshare.com/articles/dataset/geodl_example_data/23835165*](https://figshare.com/articles/dataset/geodl_example_data/23835165)*. A separate and smaller version of the data without the image chips used in the training workflow examples is also available on FigShare [48]:* [*https://figshare.com/articles/dataset/geodl_example_data_no_chips_/26824909*](https://figshare.com/articles/dataset/geodl_example_data_no_chips_/26824909)*. More general torch examples are provided on the torch website:* [*https://torch.mlverse.org/*](https://torch.mlverse.org/)*.*

The “Details of package implementation” section explains the package in detail. We feel that there is particular value in providing a detailed discussion of the implementation of the UNet architectures and associated modules, loss functions, and assessment metrics. We often struggle with the lack of clarity in implementations when using other software packages, so our goal here is to alleviate such issues for this package to aid researchers. In other words, having access to a peer reviewed article that explains the implementation in detail is of value when the package will be used in a research context.  **I installed the package from github successfully, and downloaded the scripts, but was not able to download the example data (probably because it was a 14.7 GB zip file, which can be problematic). Breaking it into smaller chunks would be more usable.**

Thanks. The software has now been published to CRAN: <https://cran.r-project.org/web/packages/geodl/index.html>

We have also built a website for the package with associated vignettes and references: <https://wvview.org/geodl/index.html>

The main size issue here arose from the inclusion of large sets of image chips to train and assess models. We have now provided a second version of the data on FigShare: one that includes the image chips and one that does not. The vignettes are now available on CRAN and also on the package website with rendered output.

We have added a paragraph to explain the sources of data and documentation in the “Need and justification” section:

*The geodl package is available on CRAN (*[*https://cran.r-project.org/web/packages/geodl/index.html*](https://cran.r-project.org/web/packages/geodl/index.html)*) and can be installed in R using install.packages(). The source code is available on GitHub (*[*https://github.com/maxwell-geospatial/geodl*](https://github.com/maxwell-geospatial/geodl)*), and the package can alternatively be installed from GitHub using the remotes package [46]. Please consult the following page for details on how to install torch and its dependencies:* [*https://torch.mlverse.org/docs/articles/installation*](https://torch.mlverse.org/docs/articles/installation)*. This page also links to documentation for configuring the environment for GPU-based computation, which is necessary to train CNN-based semantic segmentation models. The package’s official website is available at* [*https://wvview.org/geodl/index.html*](https://wvview.org/geodl/index.html)*. The package webpage complements the material presented here by providing code and detailed vignettes demonstrating the package’s workflow and associated functions. The data required to implement the examples have been provided via FigShare [47]:* [*https://figshare.com/articles/dataset/geodl_example_data/23835165*](https://figshare.com/articles/dataset/geodl_example_data/23835165)*. A separate and smaller version of the data without the image chips used in the training workflow examples is also available on FigShare [48]:* [*https://figshare.com/articles/dataset/geodl_example_data_no_chips_/26824909*](https://figshare.com/articles/dataset/geodl_example_data_no_chips_/26824909)*. More general torch examples are provided on the torch website:* [*https://torch.mlverse.org/*](https://torch.mlverse.org/)*.*

**Although I couldn't run them without data, I did look through the scripts. I note that there are hard-coded paths in various places that make them not portable. Converting these scripts to vignettes would allow for more documentation, and also help to ensure portability.**

These issues have been fixed as part of the CRAN submission and review process. Thanks.
