# Supplementary material for: geodl: An R package for geospatial deep learning semantic segmentation using torch and terra
Source: PLoS One. 2024 Dec 5;19(12):e0315127. doi: 10.1371/journal.pone.0315127 (PMC11620637; doi:10.1371/journal.pone.0315127)
Supplement: Attachment — Submitted filename: RESPONSE TO REVIEWERS.docx [file pone.0315127.s002.docx]

**RESPONSE TO REVIEWERS**

**OVERVIEW**

Thanks for reviewing our revised manuscript. Below, the reviewers’ comments are provided in **BOLD** **BLACK** text. Our comments are provided in red text. Sections extracted from the paper are *red and* *italicized*. We have tried our best to address the reviewers’ comments. We are willing to make additional edits if we are offered the chance for revision. For this revision, no updates were made to the software, software website, or example code. Only the text was edited. We did update our experiments to include a second set of experiments using ten model replicates.

Note also that we have not yet updated the associated archive paper: <https://eartharxiv.org/repository/view/7417/>. If the paper is accepted, we will update the archive version against the accepted version for consistency.

**REVIEWERS COMMENTS**

**Reviewer 1**

**Overall comments

First of all, I am a big fan of software papers, and think they need more emphasis in academia as some of the most valuable contributions researchers can make. I'm glad to see the package now available on CRAN (already with ~350 downloads since its release), resolving most of the concerns of prior reviewers.**

Thanks for reviewing our revised manuscript.  **I am coming from a remote sensing and deep learning background, although from the Python/PyTorch side of things. I have little to no experience with R or even with PLOS ONE, so forgive me if anything I say is out of touch.**

Thanks for reviewing our revised manuscript. **I really enjoyed reading this paper, especially pages 1–18 and 35–39. However, I believe pages 19–34 could be deleted without negatively impacting the quality of the paper.**

Unless the editor objects, we would prefer to maintain this section of the paper. As software users, we have found it frustrating when packages/libraries we use are not well documented by their associated reference material. The goal of these sections of the manuscript is to provide a detailed explanation of our data processing functions, UNet-based architectures, unified focal loss implementation, and assessment metrics. In other words, these sections explain the core details of our package. We feel strongly that this material is of great value to end users in documenting their projects/research. This material adds value outside of the package webpage and package documentation.

Note that we have reviewed the entire manuscript and have attempted to shorten it where possible. Our round 1 revision had a length of 13,919 words. We were able to shorten the paper to 12,946 words (i.e., we removed ~1,000 words). Instead of removing large sections of text, we instead opted to make edits throughout. Please see the edited manuscript.

**When I started reading this paper, I was really excited about learning how geodl was implemented and how it differs from other related works. However, there is very little discussion of related works other than TorchGeo. In the Python space, Raster Vision and eo-learn are also worth mentioning, while in R, the only library I could find is SITS, which sacrifices spatial support for time-series support but still involves geospatial ML.**

Thanks. We have edited the Needs and Justification section to mention these other packages/tools (i.e., Raster Vision, eo-learn, and SITS):

*“Open-source software, data science tools, and datasets have had a positive impact on how science is conducted, and the development of new tools and techniques has hastened the speed of scientific innovation and the transition of knowledge to action while also fostering reproducible and transparent research [13,14]. Open-source software and datasets have reduced cost, increased the accessibility of research tools, and become key components of research and training infrastructure. Open-source DL tools (i.e., code to support data preparation; model creation, training, and validation; and inference to new data) are currently well developed in the Python [15] language and data science environment, resulting from the development of libraries including Tensorflow [16], Keras [17], and PyTorch [18]. This can be partially attributed to the development of DL amongst the computer vision research community, wherein Python is more popular than other common data science environments [19]. Base DL libraries, such as Tensorflow and PyTorch, have been extended with additional libraries specifically focused on semantic segmentation tasks. These libraries include Kornia [20], MMSegmentation [21], PixelLib [22], and Segmentation Models [23,24]. The TorchGeo [25], Raster Vision [26], and eo-learn [27] libraries support the use of geospatial data within the PyTorch ecosystem.*

*Many data, geospatial, and Earth scientists use R [28], which was originally developed for statistical computing, data wrangling, and data analysis. This flexible environment offers a large number of specialized packages, familiarity and ease of use, quality of documentation, a large user base, and available integrated development environments (IDEs), such as RStudio [30]. Specialized R packages include those targeted for machine learning tasks such as clustering (e.g., dtwclust [31]) as well as those used for data wrangling (e.g., tidyverse [32]).*

*A large set of R packages have already been developed for reading, working with, and analyzing geospatial data specifically, such as sf [33], terra [34], stars [35], tmap [36], and sits, which supports Earth observation time series analysis [37]. The recent release of the terra package, which replaced the raster package [38] and currently (in early 2024) has had over six million downloads from the Comprehensive R Archive Network (CRAN) since its release in March 2020 based on download statistics obtained using the dlstats package [39], has improved computational efficiency for processing large raster grids, including digital elevation data and multispectral imagery [34]. Using the C++ language [40] via the rcpp package [41,42], terra allows for reading in portions of large raster grids from disk as opposed to reading the entire dataset to memory, which has greatly improved the practical application of raster-based geospatial data handling and analysis in R [34].”*

**Pages 19–34 are essentially a deep learning textbook. There are little to no novel contributions introduced here, it is simply reference material for someone looking to learn more about deep learning. I don't know what PLOS ONE papers usually look like, but I'm used to short ML conference papers that rarely ever approach 50% background material. Like the previous reviewers, it is unclear to me what the audience for this paper is. People unfamiliar with deep learning will likely need to take a machine learning course before using your model, and people familiar with deep learning already know everything on pages 19–34.**

Since this paper is not a typical research study, perhaps we did not do a good job explaining the purpose of the different sections:

Our goal with the Introduction section is to (1) outline the needs/use of the package and relate it to existing tools and (2) introduce the example data used in the case studies. So, this is similar to the Introduction section of a traditional paper.

The Overview of geodl Workflow and Details of Package Implementation sections are less Background and more Methods/Body material. The Overview of the geodl Workflow section was added based on comments from the first round of review. It is meant to provide a general overview of how the package works and the associated workflow. The Details of Package Implementation section then describes the package and its associated functions/methods in detail for those that are interested in or need to understand it in detail.

In short, we don’t see these sections as background material sections. Instead, they are meant to be part of the main body of the paper that explains the details of the package implementation. The key contributions of geodl are described including the following:

1. Provide functions to process geospatial data to create chips and associated masks/labels.
2. Allow for viewing and summarizing chips and associated masks/labels.
3. Implements a general UNet architecture that is customizable and incorporates modules that may help improve model performance (alternative activation functions, squeeze and excitation modules, attention gates, residual connections, atrous spatial pyramid pooling module, and deep supervision). This architecture draws from prior studies that have suggested augmentations of the UNet architecture. Our goal here was to aggregate some commonly used augmentations and make it easier for users to implement UNet.
4. Implement the unified focal loss framework that allows users to configure a variety of losses using a single function/class.
5. Implement common assessment metrics that are not made available by torch or terra: F1-score, precision, and recall.
6. Make it easier to validate models and calculate assessment metrics using standard methods and metrics within remote sensing and spatial predictive modeling.

In short, we feel that this material presented in pages 19-34 are essential to documenting the package. Having this material adds value, explanation, and documentation on top of that provided by the package documentation and website.  **Comments on software

In all honesty, the paper itself is more of a formality to me; the software is really what's important. In general, I don't care that much about the quality of the paper, but I want to make sure the software is well maintained and has promise.**

No comments here. Thanks. **Although the paper goes into extensive detail about the design of geodl, there is little information about its capabilities. For example, which datasets have been implemented? Which encoder and decoder model architectures? How many pre-trained model weights are available? Are all model weights pre-trained on ImageNet, or are there any actually pre-trained on remote sensing data? Is time-series data supported? Are there any plans to support classification, regression, pixelwise regression, object detection, instance segmentation, change detection, or any other common tasks in computer vision and remote sensing?**

What is currently implemented by geodl is what is outlined in the Details of Package Implementation section:

1. Utilities for creating training, validation, and testing chips and associated masks/labels. *makeMasks()* allow for creating raster masks from input vector geospatial data. *makeChips()*/*makeChipsMultiClass()* creates image chips and associated labels/mask of a defined size from larger datasets. *makeChipsDF()* lists chips and associated labels/masks into an R data frame object.
2. Utilities for viewing and obtaining summary statistics for chips and associated labels/masks: *viewChips()*, *describeChips()*, *viewBatch()*, and *descriveBatch()*.
3. A dataset subclass for reading, preprocessing, and augmenting geospatial data: *defineSegDataSet()*.
4. A general UNet architecture that is customizable and has a variety of optional modules: *defineUNet()*. Options/additional modules include defining the number of output feature maps at different stages in the architecture, alternative activation functions, squeeze and excitation modules, attention gates, residual connections, atrous spatial pyramid pooling module, and deep supervision. This flexible UNet architecture is informed from the prior studies cited throughout the paper.
5. A second UNet architecture that makes use of a MobileNet-v2 encoder backbone: *defineMobileUNet()*.
6. An implementation of the unified focal loss framework that allows users to parameterize a variety of semantic segmentation loss metrics via a single implementation: *defineUnifiedFocalLoss()*.
7. Standard semantic segmentation metrics that are not currently implemented by torch or luz: *luz_metric_overall_accuracy()*, *luz_metric_precision()*, *luz_metric_recall()*, *luz_metric_f1score()*.
8. Utilities for visualizing chip-by-chip predictions: *viewBatchPreds()*.
9. Utilities for applying trained models to infer to new geospatial data to create wall-to-wall predictions/map output: *predictSpatial()*.
10. Utilities for calculating assessment metrics using dataloaders (*assessDL()*), point locations (*assessPnts()*), and raster-based predictions/references (*assessRaster()*).

The functions, classes, models, losses, and metrics described above and within the Overview of geodl Workflow and Details of Package Implementation sections of the paper represent the functionality of geodl. Note that this is all functionality that is not made available by torch or luz. Thus, geodl adds to the torch R ecosystem and makes it easier for geospatial and remote sensing researchers/analysts that work in R to start using deep learning semantic segmentation methods in their projects/research.

We do not plan to implement any specific datasets into geodl. In the R environment, unless datasets are very small, the standard is to generate a data-only package. Instead, we plan to create additional examples that make use of different types of data. We already have examples on the package website that show how to process geospatial data into chips and associated labels/masks.

The *defineUNet()* implementation is meant to be trained from scratch. The *defineMobileUNet()* implementation can be initialized using ImageNet weights; however, it currently only accepts three-band input data.

The goal is for this package to focus on semantic segmentation. We do not plan to further expand it to other types of tasks, such as scene classification, object detection, instance segmentation, or regression.

In the Conclusions and Future Development section we explain our goals for maintaining and further expanding the package:

*“We plan to further develop geodl with future releases. First, we plan to implement a torch dataset subclass that allows for sampling from larger raster grids dynamically as opposed to generating image chips, similar to the implementation in the TorchGeo Python package [25]. We plan to implement additional CNN-based models including UNet3+ [80] and DeepLabv3+ [74,75,107]. Generally, we would like to provide a wider range of semantic segmentation algorithms and backbones, similar to the Segmentation Models Python package [23,24]. Additional development of loss functions would also be valuable, such as the ability to weight pixels based on their distance from class boundaries or select subsets of pixels for loss calculations. We would also like to expand the package to include transformer-based segmentation DL architectures, such as SegFormer [108]. We plan to develop additional functions for customizing the training loop, such as using different learning rates for different components of the model architecture. We intend for the package to maintain its focus on geospatial semantic segmentation and do not plan to implement scene classification, object detection, and instance segmentation methods. We are interested in finding others to contribute to the package. Ultimately, we hope that geodl is a useful contribution to the torch ecosystem in R.”*

We hope that our response here and our edits to the manuscript clearly explain the scope and functionality of geodl.  **I'm not familiar with R, so perhaps this is a naive question, but where is your CI/CD? Where can I find your unit tests? What percentage of the code is covered by tests? I can't, in good faith, recommend that anyone use software with less than 80% unit test coverage. For comparison, Raster Vision, eo-learn, and SITS all have over 90% unit test coverage, while TorchGeo has 100% coverage.**

For R package, the primary check or verification is getting the package accepted to the Comprehensive R Archive Network (CRAN). CRAN has strict requirements for getting a package formally accepted. This text does an excellent job explaining this process and the associated requirements: <https://r-pkgs.org/release.html>. The process includes checks of the code and associated documentations. There are both automated components and more manually checking by a member of the CRAN team. The results of the checks are available on the CRAN landing page for the package. The official CRAN page for geodl is available here: <https://cran.r-project.org/web/packages/geodl/index.html>. See the link associated with CRAN checks.

In short, we argue that the package has been exposed to a rigorous testing process via the CRAN submission.

**For the assessment metrics, it is unclear if these are part of geodl or luz. If they are not part of geodl, they shouldn't be in this paper in my opinion.**

The discussed assessment metrics (*luz_metric_overall_accuracy()*, *luz_metric_precision()*, *luz_metric_recall()*, and *luz_metric_f1score()*) are part of geodl as opposed to luz. Luz implements a few assessment metrics but also provides a more general function, *luz_metric()*, that allow users to implement new metrics that can be correctly calculated and aggregated within training and validation loops. The geodl package uses the *luz_metric()* function from luz to implement four assessment metrics that are commonly used in remote sensing and geospatial predictive modeling that are not yet implemented by torch or luz. We have tried to clarify this in the text as follows:

*“The luz package provides the luz_metric() function to allow users to define new or custom metrics for use within training and validation loops [58]. The geodl package makes use of this function to create new implementations of recall, precision, and F1-score, which are not already implemented within luz. The geodl package also includes a version of the overall accuracy (OA) metric (luz_metric_overall_accuracy()) that accepts predictions and targets defined with the shapes and data types used within the package for standardization.”*

We also clarify this in the caption for Table 5:

*“****Table 5.*** *Accuracy assessment metrics implemented by geodl using the luz_mteric() function from the luz package.”*

Note also that the unified focal loss framework is not implemented by torch or luz. Only the more commonly used classification loss metrics are implemented (e.g., binary cross entropy and cross entropy). There is no implementation of Dice or Tversky loss available in torch and luz. In short, we feel that providing the unified focal loss framework, as implemented with *defineUnifiedFocalLoss()*, is of great value as it allows users to configure a variety of losses using a single class/implementation.

**Comments on experiments

Datasets: Geodl has a number of exciting applications for remote sensing and Earth observation practitioners. However, experiments are only shown for RGB datasets, despite the vast majority of remote sensing data being multispectral, hyperspectral, thermal, or radar (SAR, inSAR). I would much rather see experiments on these data modalities, as they are more realistic for what users will actually need. Are these modalities even supported by the models you implement, or are they RGB-only?**

Our two examples make use of three-band data. The topographic maps (topoDL dataset) are interesting in that they are three-band but do not represent imagery. Instead, they represent historic cartographic information. The landcover.ai data are RGB data.

As noted in the manuscript and the examples on the geodl website, our data processing utility functions, dataset subclass, UNet-architecture implemented with *defineUNet****()***, loss metrics, assessment metrics, and inference functions can make use of variable numbers of input predictor variables or bands. We make use of the terra package to read in and work with raster geospatial data, which is not limited to three band imagery. As long as input data can be represented as raster data and associated masks can be generated, they can be used as input data to the workflow.

The only component of geodl that is currently limited to three-band data is the *defineMobileUNet()* implementation. This is due to its reliance on pre-trained encoders. In future releases, we plan to add additional architectures, including UNet3+ and DeepLabv3+, that will accepted variable numbers of input bands. This is noted in the Conclusions and Future Development section.

In the future, we plan to produce additional examples that make use of different types of data and explore different classification tasks. For example, we are currently working on examples that demonstrate extracting geomorphic features from digital terrain model-derived predictor variables.

Our choices of case studies to use in the paper and on the geodl website was primarily based on data availability.

As a quick overview of geospatial semantic segmentation datasets, there are several datasets available that focus on infrastructure. The Inria dataset (Maggiori et al., 2017) provides binary raster masks and associated high spatial resolution imagery for extracting buildings across several geographic extents: Austin, Texas, USA; Chicago, Illinois, USA; Kitsap County, Washington, USA; Vienna, Austria; and West Tyrol, Austria. The Massachusetts, USA roads and buildings datasets (Mnih, 2013) are additional examples of infrastructure-focused datasets.

The DeepGlobe datasets (Demir et al., 2018) make use of WorldView-3 imagery and represent three disparate problems: road, building, and land cover extraction.

One notable issue is that there is generally a lack of high quality, multiclass semantic segmentation datasets. We attribute this at least partially to the difficulty of generating wall-to-wall multiclass reference data. The DeepGlobe land cover (Demir et al., 2018) and landcover.ai datasets (Boguszewski et al., 2021) are notable exceptions. The landcover.ai dataset, used in one of our case studies, differentiates background, building, woodland, water, and road classes and makes use of high spatial resolution imagery for different areas in Poland. The Potsdam, Germany 2D semantic segmentation dataset (“2D Semantic Labeling Contest - Potsdam,” n.d.) consists of color infrared orthophotography, digital surface models (DSMs), and normalized digital surface models (nDSMs) at a 5 cm spatial resolution for a portion of the city. Multiple urban cover classes are differentiated, and labels are not complete.

There is also generally a lack of datasets that use predictor variables not derived from imagery or multispectral data. The vfillDL (Maxwell, 2023a), terraceDL (Maxwell, 2023b), and minebenchDL (Maxwell et al., 2024a, 2024b) datasets focus on extracting geomorphic features, valley fill faces resulting from surface coal mining, agricultural terraces for erosion control, and historic surface coal mine benches, from land surface parameters derived from digital terrain models. Again, we plan to build additional examples using some of these geomorphic-focused datasets in the future.

The topoDL dataset (Maxwell, 2024), used as one of our case studies, focuses on the extraction of surface coal mine extents from historic topographic maps, a cartographic representation of landscape characteristics as opposed to imagery.

In short, given data limitations, we feel that the landcover.ai and topoDL datasets represent good case studies for the paper and package documentation. However, we plan to add additional examples as we further develop the documentation.

**Augmentations: The experimental design is brief but riddled with mistakes. The authors choose a maximum of 1 augmentation per image, and images are only augmented 50% of the time. However, this is not how computer vision (or deep learning in general) are done. Augmentations like random horizontal and vertical flip should each be applied 50% of the time and in sequence, such that 25% are the normal orientation, 25% are horizontally flipped, 25% are vertically flipped, and 25% are both horizontally and vertically flipped. This results in the maximum possible dataset diversity, especially since overhead aerial images have no natural orientation direction. Similarly, random rotation should be applied 100% of the time, with orientations ranging from 0–360º. Modifications to things like saturation are not defined for multispectral imagery, and generally hurt performance on RGB satellite imagery where the spectral signature is important to distinguish similar classes. In the LandCover.ai experiments, the authors use: "either a vertical flip, horizontal flip, brightness adjustment, or saturation adjustment with probabilities of being applied of 0.5, 0.5, 0.1, and 0.2, respectively". However, these probabilities do not add to 1. It looks like prior reviewers also noticed this mistake, and it was fixed in the topoDL section but not the LandCover.ai section.**

We have re-executed both examples. For both examples, we have now included two experiments.

Experiment 1: Executed the model for 25 epochs using a large training set: 3,886 samples for the topoDL dataset and 4,000 samples for the landover.ai dataset. We selected the best model based on the one with the lowest loss for the validation data. In other words, the model state for the epoch that provided the best validation loss was used. This model was then used to predict to the withheld test data, generate example prediction visualizations using *viewBatchPreds()*, and calculate assessment metrics using *assessDL()*.

Experiment 2: Executed ten model replicates using different random subsets of the training chips (2,000 chips for the topoDL problem and 3,000 chips for the landcover.ai problem). We selected the best model for each replicate as the one that provided the lowest loss for the validation data. In other words, for each model replicate the model state for the epoch that provided the best validation loss was used. These models were then used to predict to the withheld test data and calculate assessment metrics using *assessDL()*. The mean, median, standard deviation, minimum, and maximum statistics were then calculated from the ten replicates and reported in tables in the text. In the text we note that this second experiment was conducted to document variability and is not meant to indicate best performance since a subset of the training chips were used and each model replicate was trained for only ten epochs.

We hope that this is adequate but are willing to further augment the experiments if necessary. However, we feel that our case studies now document/demonstrate both performance and variability via these two different experiment configurations.

We also changed how augmentations were performed. Now, up to two random augmentations, each with a 0.75 chance of being applied, were implemented. We relied only on vertical and/or horizontal flips. We did not incorporate any changes in brightness, hue, or saturation, as suggested. Note that we did not incorporate rotations since we have not implemented rotations into our *defineSegDataset()* method yet. We chose not to add rotation transformation functionality at this time due to issues of having gaps in the image extents when rotating without cropping.

The full text of the Case Studies section is provided below in response to your additional comments. **Compute: There is no mention of the computational resources used in this paper. What model of GPUs and how many GPUs were used? The model was trained for 10 epochs, but how long did that take (in hours)? This is important for comparison with other implementations to see if there are bottlenecks in the model code or in I/O.**

We have added information about our computational environment at the beginning of the Case Study section:

*“In this section, we present some sample classification results obtained using geodl. All experiments were conducted on a Windows-based workstation with an Intel i7 2.5 GHz processor, 64 GB of RAM, and a single GeForce RTX 3060 GPU with 12GB of VRAM. Two different experiments were conducted. First, models were trained for 25 epochs using a larger training set: 3,886 samples for the topoDL datasets and 4,000 samples for the landcover.ai dataset. Second, model replicates were tested using different random 2,000 sample training subsets for the topoDL problem and random 3,000 sample training subsets for the landcover.ai problem. A smaller set was used to reduce computational time and to document model variability when different random subsets of the data are used and models are initialized using different random seeds. For all experiments, the epoch that provided the lowest loss for the validation set was selected as the final model and used to predict to the withheld test data to calculate assessment metrics. When using the larger training sets, a single training and associated validation epoch took between 10 and 20 minutes to run for the topoDL problem and 1 and 1.5 hours to execute for the landcover.ai problem. We attribute the longer runtime for the lancover.ai models to the larger chip size, 512-by-512 cells as opposed to 256-by-256 cells, and the more complex model architecture that integrated the MobileNet-v2 backbone.”*

**Metrics: It's customary to provide uncertainty quantification in your reported metrics. In tables 6–8, this could be as simple as running your experiment multiple times with different PRNG seeds and reporting the mean and std dev of accuracy/precision/recall/F1. Without these, it's difficult to tell if the model always works reliably or if you just cherry-picked a good random seed.**

Please see our comments above that explain how we altered our experiments. The text of the Case studies section is provided below.

## ***Surface mine disturbance from historic topographic maps***

*Within our larger training set example for the topoDL problem, 3,886 256-by-256 cell image chips were used to train the model while 812 chips were used to validate the model at the end of each training epoch and 1,246 were maintained as a test set to assess the final model. The data were partitioned such that all chips from the same topographic map were included in the same data partition in order to avoid spatial autocorrelation between the data partitions. For the training set, a maximum of two augmentations were performed per chip, vertical or horizontal flips, with a 0.75 probability of being applied. In other words, up to two augmentations were applied to a single chip, and there was a 75% chance of applying each augmentation. If no augmentation is applied, the original chip is returned. A mini-batch size of 15 was used, and the model was trained for 25 epochs. The model that returned the lowest loss for the validation data was maintained as the final model, which was the model state after epoch 20. The modified unified focal loss was configured as a Tversky loss using λ = 0, γ = 1, and δ = 0.6. In other words, only the region-based loss was included with no focal adjustment but unequal weighting for FN and FP errors per class. The AdamW optimizer was used with a learning rate of 1e-3, which was selected using the learning rate finder implementation in the luz package.*

*Table 6 provides the confusion matrix and derived metrics calculated for the withheld test set and using the assessDL() function while Fig 11 provides an example set of image chips (a), reference masks (b), and predictions (c) for a mini-batch of test samples created using the viewBatchPreds() function. The OA for the prediction was 0.990 while the F1-score was 0.963. The precision was 0.954 while the recall was 0.971, suggesting a good compromise between commission and omission errors. Table 7 shows summary statistics for the assessment metrics calculated using ten model replicates, different random seeds, and different random subsets of 2,000 training chips. Again, this second experiment was conducted to document performance variability and is not meant to indicate best performance since a subset of the training chips were used and each model replicate was trained for up to only ten epochs.*

***Table 6.*** *Confusion matrix and derived metrics for topoDL [52] classification. Values represent counts of pixels or cells. OA = overall accuracy; NPV = negative predictive value.*

|  |  | ***Reference*** | |  |
| --- | --- | --- | --- | --- |
|  |  | *Background* | *Mine* |  |
| ***Prediction*** | *Background* | *69,762,584* | *326,278* | *NPV = 0.995* |
|  | *Mine* | *527,709* | *10,975,749* | *Precision = 0.954* |
|  |  | *Specificity = 0.993* | *Recall = 0.971* | *F1-Score = 0.963*  *OA = 0.990* |

*[INSERT FIGURE 11]*

***Fig 11.*** *Example prediction of surface mine disturbance extents from topoDL dataset [52]. (a) example image chips; (b) reference labels or masks, (c) predictions generated using geodl workflow.*

***Table 7.*** *Model assessment metrics based on ten model replicates with different random seeds and training subsets. OA = overall accuracy; NPV = negative predictive value.*

|  | ***OA*** | ***F1-score*** | ***Recall*** | ***Precision*** | ***Specificity*** | ***NPV*** |
| --- | --- | --- | --- | --- | --- | --- |
| *Mean* | *0.981* | *0.930* | *0.938* | *0.923* | *0.987* | *0.990* |
| *Median* | *0.983* | *0.937* | *0.941* | *0.935* | *0.990* | *0.991* |
| *SD* | *0.004* | *0.014* | *0.023* | *0.023* | *0.004* | *0.004* |
| *Minimum* | *0.972* | *0.903* | *0.892* | *0.864* | *0.976* | *0.983* |
| *Maximum* | *0.985* | *0.947* | *0.968* | *0.940* | *0.991* | *0.995* |

## ***Landcover.ai multiclass land cover classification***

*For the landcover.ai experiment, 4,000 512-by-512 cell image chips were used to train the model while 1,000 chips were used to validate the model at the end of each training epoch and 792 were maintained as a testing set to assess the final model. Data partitions were defined by the data originators. For the training set, a maximum of two augmentations were performed per chip, vertical or horizontal flips, with a probability of 0.75 of being applied. A mini-batch size of 20 was used, and each model was trained for 25 epochs. The model that returned the lowest loss for the validation data, the model state after epoch 21, was maintained as the final model. The modified unified focal loss was configured as a focal Dice loss using λ = 0, γ = 0.8, and δ = 0.5. In other words, only the region-based loss was included, a focal adjustment was applied to increase the relative cost of misclassifying difficult classes, and FN and FP errors per class were equally weighted. The AdamW optimizer was used with a learning rate of 1e-3, which was selected using the learning rate finder implementation in the luz package.*

*Table 8 provides a confusion matrix, class-level user’s and producer’s accuracies, and class-level F1-scores calculated for the withheld test set and using the assessDL() function while Fig 12 provides an example set of image chips (a), reference masks (b), and predictions (c) for a mini-batch of testing samples created using the viewBatchPreds() function. The OA was 0.908 and the macro-averaged, class-aggregated F1-score was 0.823. The woodland class showed both the lowest producer’s and user’s accuracies. Table 9 provides the OA and macro-averaged, class-aggregated F1-score (aF1), producer’s accuracy (recall) (aPA), and user’s accuracy (precision) (aUA) summary metrics calculated for the ten model replicates using different random seeds and 3,000-sample subsets of the available training chips.* *Again, this second experiment was conducted to document variability and is not meant to indicate best performance since a subset of the training chips were used and each model replicate was trained for only ten epochs.*

***Table 8.*** *Confusion matrix and class-level user’s and producer’s accuracies for landcover.ai [53] classification. Overall accuracy = 0.908, macro-averaged producer’s accuracy = 0.885, macro-averaged user’s accuracy = 0.770, and macro-averaged F1-score = 0.823.*

|  |  | ***Reference*** | | | | |  |
| --- | --- | --- | --- | --- | --- | --- | --- |
|  |  | *Background* | *Building* | *Woodland* | *Water* | *Road* | *User’s Accuracy* |
| ***Prediction*** | *Background* | *102,511,856* | *197,898* | *381,378* | *561,041* | *2,120,744* | *0.969* |
|  | *Building* | *588,494* | *1,843,051* | *23,588* | *3,089* | *5,063* | *0.748* |
|  | *Woodland* | *4,356,259* | *46,980* | *2,984,637* | *15,177* | *153,713* | *0.395* |
|  | *Water* | *1,386,604* | *13,854* | *27,477* | *9,723,005* | *295,632* | *0.849* |
|  | *Road* | *8,400,555* | *27,141* | *402,337* | *86,647* | *71,461,828* | *0.889* |
|  | *Producer’s Accuracy* | *0.874* | *0.866* | *0.781* | *0.936* | *0.965* |  |
|  | *F1-score* | *0.919* | *0.803* | *0.525* | *0.891* | *0.926* |  |

*[INSERT FIGURE 12]*

***Fig 12.*** *Example prediction of general land cover from landcover.ai dataset [53]. (a) example image chips; (b) reference labels or masks, (c) predictions generated using geodl workflow.*

***Table 9.*** *Overall accuracy and macro-averaged class aggregated assessment metrics for landcover.ai [53] classification using ten replicates and different 3,000 chip random data partitions. OA = overall accuracy, aF1 = macro-averaged, class aggregated F1-score, aPA = macro-averaged, class aggregated producer’s accuracy (recall), aUA = macro-averaged, class aggregated user’s accuracy (precision).*

|  | ***OA*** | ***aF1*** | ***aPA*** | ***aUA*** |
| --- | --- | --- | --- | --- |
| *Mean* | *0.921* | *0.829* | *0.816* | *0.843* |
| *Median* | *0.922* | *0.831* | *0.824* | *0.847* |
| *SD* | *0.004* | *0.020* | *0.021* | *0.025* |
| *Minimum* | *0.915* | *0.774* | *0.763* | *0.786* |
| *Maximum* | *0.928* | *0.852* | *0.836* | *0.881* |

**Comments on writing

The overall writing quality is extremely high and practically devoid of typos and grammar mistakes. My only complaint is the percentage of the paper that feels like well-known background material that can be found in any deep learning textbook.**

We have no additional comments here. We have justified our Overview of geodl Workflow and Details of Package Implementation sections above. Again, we argue that these section represent core content and provide key explanations of the provided implementations.  **I would add more detailed captions to all figures and tables. Each figure and table should be standalone and self-explanatory, even without the surrounding text.**

We have revised the table and figure captions. Please see the edited text.  **Table 1: I would change the purpose of "validation data" from "assess model generalization" to "evaluate hyperparameter choices". Again, all of these definitions are standard. I don't think it's possible to pass a single ML course without knowing these.**

We edited this row of Table 1 as suggested.

| Validation data | Data partition of chips and associated masks which are commonly predicted at the end of each training epoch; used for hyperparameter tuning. |
| --- | --- |

**Figure 1/2: The diagram makes it look like geodl only supports vector masks. It may be good to clarify whether or not geodl also supports raster masks, and update the diagram to show multiple possible starting points if that is the case.**

Thanks. We clarified this by editing the figure captions as follows:

*“****Fig 1.*** *Overview of geodl workflow. Note that raster-based masks or labels can be used. The makeMasks() function is provided to convert vector-based reference data into raster masks.”*

*“****Fig 2.*** *Conceptualization of geodl DL semantic segmentation workflow. Functions prefixed with torch:: are from the torch package while those prefixed with luz:: are from the luz package. All other functions are from geodl. Functions with a green check mark indicate those that are used as checks during the workflow or to assess trained models. Diamonds indicate outputs or results: the trained model and predictions to new raster data. Note that raster-based masks or labels can be used. The makeMasks() function is provided to convert vector-based reference data into raster masks.”*

**Equation 3: This equation is not in parentheses, unlike all other equations.**

The parentheses in Equation 1 and 2 are necessary since there are different calculations based on conditions (i.e., whether or not the activation is lower than or greater than or equal to 0). Since Equation 3 does not have a condition, we don’t think parentheses are necessary.

**Reviewer 2**

**Thank you for the impressive amount of effort you put into not just preparing your package to pass CRAN checks and requirements, but also in developing multiple vignettes, example datasets, and clear documentation.

From my perspective as an experienced R user with interest in but no experience with geospatial DL, I found the reorganized manuscript much easier to follow. Clearly separating the sections into general overview and more technical material, and adding more explanation (like Table 1), was very helpful for me. Figures 1 and 2 support this organization, with both high-level and more detailed workflows that clearly denote functions included in this package vs those from other important packages.

This is a challenging but very hot topic, and I think that your package will be of great interest.**

Thanks for reviewing our revised manuscript.

**Reviewer 3**

**The manuscript is a resubmission of the authors' previous work. In this revised version, the authors have improved the clarity of the manuscript, published the R package on CRAN, and satisfactorily addressed the issues raised by my previous review.**

Thanks for reviewing our revised manuscript.

**Reviewer 4**

**This paper introduces a R package for training deep learning semantic segmentation models with geospatial data and shows two applications of this package with existing benchmark datasets from the literature. This is a commendable and important effort that fills a large gap in functionality in the R ecosystem -- enabling researchers in scientific fields where R is a standard to work with modern ML approaches for semantic segmentation. Modern semantic segmentation models, along with the necessary training and testing pipelines to use them are non-trivial to implement correctly (further, when implemented incorrectly they can still seem to work for many reasons). Scientific users should be abstracted away from the details of implementation in order to focus on their specific problem and this cross-cutting infrastructure work is important to making this happen. The acceptance of the package on CRAN is a great milestone for this work!**

Thanks for reviewing our revised manuscript.

**From a results standpoint, the experiments on LandCoverAI show performance that is similar to other approaches reported in the literature. The experiments on the surface mines give a strong benchmark that other methods can attempt to beat and that represent a usable approach to detecting surface mines in practice.**

Thanks for reviewing our revised manuscript.

**My only minor comments would be to condense the writing where possible (the current manuscript is very long), consider adding space or larger borders between patches in Fig 3, 4, 11, 12, consider removing the architecture diagrams in favor of citing the original work (I'm not sure these improve understanding of the method or help make a point other than, "the models are non-trivial to implement"), and consider a careful proofreading (e.g. in the abstract I might edit, "Such methods are especially applicable to pixel-level classification, or semantic segmentation, tasks." --> "Such methods are especially applicable to pixel-level classification or semantic segmentation tasks.").**

Thanks for reviewing our revised manuscript.

We have reviewed the entire manuscript and have attempted to shorten it where possible. Our round 1 revision had a length of 13,919 words. We were able to shorten the paper to 12,946 words (i.e., we removed ~1,000 words). Instead of removing large sections of text, we instead opted to make edits throughout. Please see the edited manuscript.

We have added white grid lines to Fig. 3, 4, 11, and 12 to make the individual chips more distinguishable.

Unless the editor objects, we would prefer to maintain all of our architectural diagrams so that the paper fully documents the UNet implementation provided by the package. One of the primary goals of this paper is to serve as a detailed reference to the package and implemented architectures. We have found it frustrating in the past when architectures and/or software that we have used in our own research/projects are not well documented by their associated publications and/or reference material. In short, we feel that there is merit in including the diagrams to provide a single, detailed explanation of the UNet architecture to aid users in implementing it and understanding it more fully.

We edited the sentence in the abstract as suggested and reviewed the entire manuscript. Please see the edited manuscript that documents the editing performed.
